# Introduction of robotic surgery for endometrial cancer into a Brazilian cancer service: a randomized trial evaluating perioperative clinical outcomes and costs

**DOI:** 10.6061/clinics/2017/e522s

**Published:** 2018-09-11

**Authors:** Alexandre Silva e Silva, João Paulo Mancusi de Carvalho, Cristina Anton, Rodrigo Pinto Fernandes, Edmund Chada Baracat, Jesus Paula Carvalho

**Affiliations:** Disciplina de Ginecologia, Instituto do Cancer do Estado de Sao Paulo (ICESP), Hospital das Clinicas HCFMUSP, Faculdade de Medicina, Universidade de Sao Paulo, Sao Paulo, SP, BR

**Keywords:** Endometrial Cancer, Robotic Surgery, Laparoscopic Surgery, Lymph Node Dissection, Surgery

## Abstract

**OBJECTIVE:**

The purpose of this study was to evaluate the clinical outcome and costs after the implementation of robotic surgery in the treatment of endometrial cancer, compared to the traditional laparoscopic approach.

**METHODS:**

In this prospective randomized study from 2015 to 2017, eighty-nine patients with endometrial carcinoma that was clinically restricted to the uterus were randomized in robotic surgery (44 cases) and traditional laparoscopic surgery (45 cases). We compared the number of retrieved lymph nodes, total time of surgery, time of each surgical step, blood loss, length of hospital stay, major and minor complications, conversion rates and costs.

**RESULTS:**

The ages of the patients ranged from 47 to 69 years. The median body mass index was 31.1 (21.4-54.2) in the robotic surgery arm and 31.6 (22.9-58.6) in the traditional laparoscopic arm. The median tumor sizes were 4.0 (1.5-10.0) cm and 4.0 (0.0-9.0) cm in the robotic and traditional laparoscopic surgery groups, respectively. The median total numbers of lymph nodes retrieved were 19 (3-61) and 20 (4-34) in the robotic and traditional laparoscopic surgery arms, respectively. The median total duration of the whole procedure was 319.5 (170-520) minutes in the robotic surgery arm and 248 (85-465) minutes in the traditional laparoscopic arm. Eight major complications were registered in each group. The total cost was 41% higher for robotic surgery than for traditional laparoscopic surgery.

**CONCLUSIONS:**

Robotic surgery for endometrial cancer presented equivalent perioperative morbidity to that of traditional laparoscopic surgery. The duration and total cost of robotic surgery were higher than those of traditional laparoscopic surgery.

## INTRODUCTION

Endometrial cancer is the eighth most common cancer in Brazilian women, with 6,950 new cases estimated for 2016 at an incidence of 6.74 cases per 100,000 women. Endometrial cancer is more frequent in the southeast region of Brazil (9.58 cases/100,000) 1. Surgery is still considered the main treatment for endometrial cancer. Most surgeries in Brazil are still performed by gynecologists and obstetricians in general hospitals, and most of these clinicians perform only laparotomic surgeries.

Since 2009, at the Gynecological Division of the *Instituto do Câncer do Estado de São Paulo (ICESP)*, endometrial cancer has been preferably treated by laparoscopy, and treatment has followed the recommendations of FIGO, namely, complete staging surgery comprising removal of the uterus, ovaries, uterine tubes, and pelvic and paraaortic lymph nodes 2. Robotic surgery using the da Vinci^®^ robot (Intuitive Surgery Inc., CA, USA) was introduced to our institution in 2015 as a research project and has since been used for endometrial cancer staging. We aimed to evaluate the perioperative advantages, disadvantages and costs of robotic surgery compared to traditional laparoscopic surgery in patients with endometrial cancer.

## METHODS

Patients with endometrial cancer at the ICESP and candidates for primary surgical treatment were prospectively identified through the hospital's electronic medical record. From January 2015 to June 2017, ninety consecutive patients with endometrial cancer that was apparently restricted to the uterus and candidates for minimally invasive surgeries were randomized to robotic or traditional laparoscopic surgical arms. Two experienced laparoscopic surgeons performed both the robotic and the traditional laparoscopic surgeries (ASS and JPMC). The study was approved by the center's Institutional Review Board, and written informed consent was provided by all patients (number of protocol CEP-FMUSP 438/13).

The method of randomization was as follows: we used the randomization of the permutated block to allow an adequate random distribution of patients between the groups. A function was created in Microsoft Excel (MS Excel), with randomization between blocks of 6, 8 and 10, and a list was generated with a sequence of 100 numbers. The randomization list was password-protected and was the responsibility of the study nurse. The sequence of numbers was hidden; that is, the research nurse had access to the patient's random number only after signing the ICF (inclusion in the study) and inserting the data into the worksheet. The surgeons participating in the project did not have access to the spreadsheet. The research nurse utilized email and performed insertions of information into institutional electronic medical records to inform the study team of the group in which the patient was allocated.

The following data were collected from the electronic medical record: age, body mass index, histological type, histological grade, tumor size, stage, number of pelvic lymph nodes, number of paraaortic lymph nodes, total duration of surgery, and the durations of several procedures, namely, right pelvic lymphadenectomy, left pelvic lymphadenectomy, paraaortic lymphadenectomy, hysterectomy and closure of the vaginal cuff.

In the traditional laparoscopic surgery arm, we used conventional permanent instruments from Karl Storz^®^ (Stuttgart, Germany), with a high-definition camera and disposable advanced energy devices LigaSure^®^-Medtronic (Minneapolis, MN, USA) or Ultracision^®^ (Ethicon Endo-Surgery Inc., Cincinnati, OH, USA). All patients had previous clinical and anesthetic evaluations, radiological evaluations (thoracic and abdominal computed tomography and magnetic resonance imaging of the pelvis).

In all but two patients, single docking was used. Robotic surgery cases were performed with a supraumbilical 13-mm port for the camera and three 8-mm operating arms disposed in the arch. Another 12-mm, and sometimes another 5-mm port, was placed on the right upper abdominal quadrant for use of the assistant. At the end of the surgery, all specimens were retrieved through the vagina. In six patients, the specimens were removed via a Pfannenstiel incision because of an enlarged uterus. Traditional laparoscopic surgeries were performed with two 5-mm ports close to the anterior iliac spine bilaterally and two 12-mm ports located at the umbilicus and suprapubic region. In all patients, the surgical plan was to remove the uterus, ovaries, uterine tubes, and pelvic and paraaortic lymph nodes. The upper anatomical limit of the dissection of the paraaortic lymph nodes was the level of the left renal vein.

The medians were compared with the Kruskal-Wallis test. The categorical variables were compared with the chi-square test. Statistical analyses were performed using MedCalc for Windows (version 17.9.2; MedCalc Software, Mariakerke, Belgium), and p-values less than 0.05 were considered significant.

The da Vinci^®^ robot was donated to our hospital by the Brazilian government's Ministry of Science and Technology to carry out a multidisciplinary project. The aim was to evaluate the advantages and disadvantages of robotic surgery in a public hospital for the treatment of cancer in various specialties such as urological, digestive, head and neck, thoracic and gynecological surgeries.

The total cost to the hospital did not include the cost of the robot itself but did include the costs of hospital admission, theaters, drugs and pharmacy, blood products, high-dependence care, imaging, pathology, medical staffing and rehabilitation therapy. Cost data were analyzed without considering the cost of the robot acquisition.

In this study, we analyzed the perioperative outcomes and costs of the patients with endometrial cancer treated by robotic surgery versus traditional laparoscopic surgery.

## RESULTS

Eighty-nine patients were randomized. Two candidates in the robotic arm were excluded (one patient refused surgical treatment for religious reasons, and the other patient abandoned treatment). In the traditional laparoscopic arm, two patients were excluded (one because she had developed inoperable peritoneal carcinomatosis and another due to poor clinical condition related to morbid obesity (BMI = 58.6 kg/m^2^)).

The patients who were distributed between the two arms of the study were similar according to age, BMI, preoperative histology, tumor grade, tumor size, and FIGO stage (Table 1).

The median total number of lymph nodes retrieved was 19 (3-61) in the robotic surgery arm and 20 (4-34) in the traditional laparoscopic arm. The median numbers of retrieved paraaortic lymph nodes were 11.5 (0-32) and 15 (0-41) in robotic and traditional laparoscopic arms, respectively.

In our study, robotic surgery was more time consuming than traditional laparoscopic surgery. The median total duration of the whole procedure was 319.5 (170-520) minutes in the robotic surgery arm and 248 (85-465) minutes in the traditional laparoscopic surgery. We also separately analyzed the time in minutes devoted to each of the following procedures: right pelvic lymphadenectomy, 44.5 (26-128) *vs.* 33 (21-77); left pelvic lymphadenectomy, 43.5 (27-110) *vs.* 31 (18-59); paraaortic lymphadenectomy, 93 (24-139) *vs.* 77 (40-115); hysterectomy, 32 (15-62) *vs.* 23 (8-62); and vaginal closure, 14 (7-39) *vs.* 15.5 (9-40). The estimated blood loss was 162 (0-2915) ml and 105.5 (0-1465) ml in the robotic and traditional laparoscopic surgical arms, respectively.

The median hospital stay was three days and was similar in both groups. One patient in the traditional laparoscopic surgery arm remained hospitalized for forty-three days until death due to septicemia. This patient had an infected and necrotic grade 3 endometrioid carcinoma. In the robotic group, a death occurred due to an unnoticed perforation of the duodenum. This patient developed peritonitis, and autopsy examination confirmed perforation of the duodenum.

In the robotic surgery arm, there was one conversion to laparotomy to correct a vena cava lesion, while in the traditional laparoscopic arm, there were two conversions to laparotomy, one due to advanced disease and another for multiple peritoneal adhesions.

There were eight occurrences of major complications in each arm. Major complications included vena cava, duodenal, obturator nerve, iliac artery and ureter injuries. Other major complications included cases of thromboembolism and sepsis. Minor complications occurred in six cases in the robotic surgery arm and in two cases in the traditional laparoscopic arm. These complications comprised two cases of urinary tract infection, two cases of hernia in the trocar sites, one case of panniculitis, one case of vaginal cuff dehiscence, one case of bladder injury and one case of intestinal subocclusion. The most severe of all perioperative complications was vena cava injury. This complication occurred three times in both the robotic and traditional laparoscopic arms.

### Costs analysis

The following costs were considered: the daily cost of hospitalization, intensive care unit admission, disposable material, medication, surgical theaters (per minute), medical gases (per minute), robot instruments; and therapeutic diagnostic and support services (Table 2). The cost of reusable robot instruments was calculated considering that each instrument could be used in 10 procedures. The standard staff for both types of surgery consisted of three surgeons, an anesthesiologist, a circulating nurse, and a scrub nurse.

Statistical analysis of costs was performed using the software SPSS version 19 (SPSS, Inc, Chicago, IL, USA). The total costs were compared between the two surgery groups. For the comparison between the two groups, the U Mann-Whitney test was used. The chi-square test was used for nominal variables. The estimated total costs and subcategories (in US dollars) of the surgeries performed by robot versus laparoscopy are presented in Table 2.

Without considering the acquisition and maintenance costs of the robot, the estimated median total costs for each endometrial cancer surgical treatment in our institution was 6,812 US dollars (SD ± 1849) for traditional laparoscopic surgery and 9,655 US dollars (SD ± 850) for robotic surgery (*p*<0.001).

## DISCUSSION

With the advent of minimally invasive methods, surgery for endometrial cancer has evolved substantially in recent decades 3. One of the major advancements was the change from open surgery to laparoscopic surgery that occurred in the late 1990s and that resulted in lower perioperative morbidity without losing the radicality or effectiveness of oncological surgery 4-6.

However, the high complexity of traditional laparoscopic procedures requires time-consuming training. Different studies have shown that robotic hysterectomy with lymphadenectomy has a shorter learning curve than the laparoscopic approach 7 and that robotic surgery can be a suitable alternative to compensate for the lengthy training time needed to qualify the gynecological oncology surgeon for laparoscopic surgery.

In our current study, there were no significant differences between the patients subjected to robotic and traditional laparoscopic surgery in relation to age, body mass index, histological type, histological grade, tumor size, tumor stage or the total number of lymph nodes retrieved. Obesity poses a major challenge in laparoscopic surgery for endometrial carcinoma, and each additional unit of body mass index increases the risk of failure in complete laparoscopic surgery by 11% 8. Two-thirds of our patients had a body mass index greater than thirty. In the population served at our institution, endometrial carcinoma presents in more advanced stages 2. Only 14% of our patients had a tumor smaller than 2 cm.

The median total surgical time was higher in the robotic surgery group, at 319.5 (170-520) minutes, than in the traditional laparoscopic surgery group, at 248 (164-465) minutes. Leitao et al. 9 reported that robotic surgery requires the same amount of time as laparoscopic surgery until completion of the learning curve, which is considered to occur with forty cases.

Regarding the number of lymph nodes removed, Chan et al. 10 reported that in high-risk and intermediate-risk endometrial cancer patients, 5-year survival was progressively higher according to the number of lymph nodes removed (75.3%, 81.5%, 84.1%, 85.3%, and 86.8% survival for 1, 2-5, 6-10, 11-20, and >20 lymph nodes, respectively).

In another study, Cragun et al. 11 found that patients with undifferentiated tumors with 11 pelvic lymph nodes removed had a better overall survival rate than patients with less than 11 pelvic lymph nodes removed. In our study, the number of lymph nodes retrieved was equivalent in both types of surgery, namely, 29.5 (10-93) in robotic surgery and 34 (5-70) in traditional laparoscopic surgery, which was considered sufficient.

The number of serious complications was expected to be higher at the beginning of the learning curve. We observed the same major complication rates when robotic surgery was compared with traditional laparoscopic surgery. However, when we compared the results of the first and second half of the study, we observed a marked decline in major complications (11 *vs*. 5), as well as minor complications (6 *vs*. 2), in the second half of the study. For the laparoscopic surgeries, our two surgeons had already completed the learning curve, while for robotic surgery, they were just beginning the learning curve. We did not evaluate the outcomes of robotic surgery performed by surgeons who were not experienced in endoscopic surgery.

We regarded any vena cava injury as a serious complication regardless of the extent and consequences for the patient. Vena cava injuries occurred three times in each type of surgery. In all but one case, the lesion was repaired without conversion to laparotomy. Injury to the vena cava at the time of the paraaortic lymphadenectomy is an event that has been reported in different series since the beginning of the era of minimally invasive surgery 12-14. One patient had unperceived duodenum perforation. In the immediate postoperative period, this patient developed peritonitis and septic shock and subsequently died.

Sectioning of the obturator nerve at the time of pelvic lymphadenectomy is a complication that has been reported in some series 15,16. We observed sectioning of the obturator nerve in one robotic surgery and in two traditional laparoscopic surgeries. These injuries were successfully repaired without conversion to laparotomy. Other complications included two cases of ureter perforation, one case of iliac artery perforation and two cases of thromboembolism.

The major obstacle to the use of robotic surgery in the treatment of endometrial cancer is the cost of the system. The decision to implement robotic surgery in a public healthcare system should consider the total cost to the institution, which includes the costs of the robot, hospital admission, theaters, drugs and pharmacy, blood products, high-dependence care, imaging, pathology, medical staff and rehabilitation therapy.

The total cost of robotic surgery depends on multiple factors that vary between different countries: the type of hospital, namely, general hospitals versus referral centers; the volume of surgeries performed; the previous experience of the team in minimally invasive surgeries; the cost of disposable materials; the cost of the use of the different surgical instruments; the duration of operating room use; the use of medical gases and medicines; and the cost of the team of medical professionals and paramedics involved. Costs associated with patient rehabilitation and treatment of complications may also be included. For these reasons, the economic feasibility studies of robotic surgery present different results and need to be considered within the reality of each institution. There are several publications with economic evaluations of the implementation of robotic hysterectomy compared to the implementation of laparoscopic or open surgery. Many of these results should not be generalized because they compare different cost categories. The costs of robotic surgery compared to laparoscopic or open surgery vary greatly in different studies. A comparison of the estimated cost of robotic hysterectomy (in US dollar) in different countries are presented in table 3 17-22.

Approximately seven hundred cancer surgeries are performed every month in our institution, and there is only one robot to be shared between different specialties. The robot was provided by the government. Since 2009, the surgical treatment of choice for endometrial carcinoma in our hospital has been traditional laparoscopic surgery. The use of the robot was not an option for surgeons in general. The robot was only available for cases included in research protocols involving all surgical specialties and for protocols that had evaluations of the impact of robotic surgery on patient outcomes and economic viability in the institution as their research objectives. An analysis of the costs of each procedure will be carried out in the future, along with an analysis of all specialties.

The costs of the robot-specific supplies are the main drivers of additional costs compared with traditional laparoscopic surgery. In our study, robotic hysterectomy for the treatment of endometrial cancer was 41.7% more expensive than traditional laparoscopic surgery and had an equivalent perioperative outcome.

Despite the variations in the absolute values of costs in different countries, we can clearly state that robotic surgery is still more expensive than traditional laparoscopic surgery, and the justification for its introduction into an institution is still based on reasons other than costs.

One of the most relevant indirect advantages of robotic surgery is its ability to allow institutions with a low volume of minimally invasive surgeries to change this profile by introducing robotic surgery that requires less time to complete the learning curve. Lau et al. 23 have reported that the rate of minimally invasive surgeries rose from 17% to 98% with the introduction of robotic surgery. In our institution, most surgeries for endometrial carcinoma (62%) are performed by laparoscopy 2, and the incorporation of the robot did not have a great impact on the number of patients treated by minimally invasive surgery. We have only one robot that is shared by surgeons of all other specialties in addition to gynecological oncology. This fact represents a limitation for the use of the robot for a small number of patients.

The introduction of robotic surgery in our public hospital for the treatment of endometrial cancer demonstrated perioperative morbidity that was equivalent to that of the traditional laparoscopic surgery performed by the same surgeons. The duration of robotic surgery was higher than that of traditional laparoscopic surgery. The total cost of robotic surgery was 41% higher than that of traditional laparoscopic surgery at our institution. Incorporation of the robot did not have a great impact on the number of patients treated by minimally invasive surgery because we have only one robot that is available to a small number of patients.

## AUTHOR CONTRIBUTIONS

Silva e Silva A and Carvalho JP conceptualized the study, participated in the design of the manuscript and in the draft of the manuscript, and carried out half of the surgeries. Anton C and Fernandes RP participated in the design of the manuscript and coordinated the study. Baracat EC provided critical revisions to the manuscript. Carvalho JP conceptualized the study and participated in the design, draft and writing of the manuscript.

## Figures and Tables

**Table 1:** Patient characteristics according to surgery type.

Type of surgery (no.)	Robotic (42)	Traditional laparoscopic (43)	*p*
Age at surgery (average)	60 (47-69)	60 (48-69)	0.36
BMI category	31.7 (21.4-54.2)	30.3 (24.8-42.7)	0.43
Preoperative histology	Endometrioid (37)	Endometrioid (37)	0.77
	Non-endometrioid (5)	Non-endometrioid (6)	
Grade	Low (32)	Low (29)	0.20
	High (8)	High (14)	
Size (cm-average)	4.0 (1.5-10.0)	4.0 (0.0-9.0)	0.41
FIGO stage	IA (17)	IA (18)	0.44
	IB (13)	IB (16)	
	II (4)	II (1)	
	IIIA (2)	IIIA (1)	
	IIIC1 (2)	IIIC2 (5)	
	IIIC2 (2)	IVB (2)	
	IVB (2)		
Total number of lymph nodes	29.5 (10-93)	34 (5-70)	0.36
Paraaortic lymph nodes	11.5 (0-32)	15 (0-41)	0.08
Pelvic lymph nodes	19 (3-61)	20 (4-34)	0.72
Total length of surgery (minutes)	319.5 (170-520)	248 (164-465)	0.000042
Length (minutes)			
Right pelvic	44.5 (26-128)	33 (21-77)	
Left pelvic	43.5 (27-110)	31 (18-59)	
Paraaortic	93 (24-139)	77 (40-115)	
Hysterectomy	32 (15-62)	23 (8-62)	
Vaginal closure	14 (7-39)	15.5 (9-40)	
Estimated blood loss (ml)	162 (0-2915)	105.5 (0-1465)	0.64
Hospital stay (days)	3 (2-5)	3 (2-43)	0.78
Perioperative death	1	1	
**Total major complications**	8	8	0.96
Vena cava injury	3	3	
Duodenal injury	1	0	
Obturator nerve injury	1	2	
Iliac artery injury	1	0	
Ureter injury	1	1	
Thromboembolism	1	1	
Sepsis	0	1	
**Total minor complications**	6	2	0.97
Urinary tract infection	2	0	
Trocar site hernia	1	1	
Panniculitis	1	0	
Vaginal cuff dehiscence	1	0	
Bladder injury	0	1	
Intestinal subocclusion	1	0	
Conversion to laparotomy	1	2	0.31

**Table 2:** Estimated costs (in US dollars) of surgeries performed by robot *versus* laparoscopy.

	Laparoscopies (n = 42)				Robotic surgeries (n = 43)				
	Average	SD	Min.	Max.	Average	SD	Min.	Max.	*p* value*
Daily cost of Hospitalization	495.1874	359.6528	317.5251	2381.4354	492.8465	166.2634	316.3032	1107.0657	0.427
Daily cost of ICU	56.0677	147.0435	0.00	672.8136	78.2341	161.4552	0.00	672.8136	0.437
Material	897.6983	90.4122	755.0503	1090.8152	1462.3576	314.5286	600.7796	2144.1609	<0.001
Medication	259.2113	246.2943	36.6032	889.4416	243.1237	99.3109	54.3573	412.0495	0.068
Surgical room (per minute)	2169.2138	407.6188	443.1585	3066.2954	2192.1730	736.5384	1148.3991	4135.8144	0.209
Medical gas (per minute)	36.2652	11.2955	5.4447	63.3009	107.5381	137.9227	22.4625	647.2885	<0.001
OPSM	577.2401	238.6439	38.2666	1244.7133	2155.9497	177.3411	1840.8357	2592.0006	<0.001
TDSS	97.1602	97.9050	5.2715	507.0626	112.5827	122.0792	7.6442	665.6272	0.571
Employment-personal	2224.6055	323.2454	1742.6667	3209.6644	2810.3044	868.1820	1472.3001	5391.1307	<0.001
**Total Cost**	**6812.6498**	**850.1143**	**5210.6234**	**8856.8415**	**9655.1099**	**1849.3956**	**6524.6404**	**14911.9969**	**<0.001**

* Mann-Whitney test;

SD: Standard deviation;

OPSM: Orthosis, Prostheses and Special Materials;

TDSS: Therapeutic Diagnostic Support Service.

**Table 3:** Comparison of estimated cost of robotic hysterectomy (in US dollars) in different countries.

	RH	LH	OH	Historical	Country
Pellegrini et al. 18	4.281	1.085	1.301		Italy
Lau et al. 17	6.028			8.176	Canada
Nitschmann et al. 19	3.150				USA
Coronado et al. 20	6.006	5.466	5.569		Spain
Salehi et al. 21	19.937		21.505		Sweden
Fader et al. 22	12.408	10.809	10.935		USA
Sarlos et al. 23	4.839	2.151			Swiss

RH = robotic hysterectomy;

LH = laparoscopic hysterectomy;

OH = open hysterectomy.
